# A Risk-Difference Meta-Analysis for the Prophylactic Treatments of Chronic Migraine

**DOI:** 10.7759/cureus.62458

**Published:** 2024-06-16

**Authors:** Michalis Kodounis, Theodoros S Constantinidis, Konstantina Rizonaki, Eleni Drakou, Elias Zintzaras, Ioannis Stefanidis, Dimos-Dimitrios Mitsikostas, Efthimios Dardiotis

**Affiliations:** 1 First Neurology Department, Eginitio Hospital, School of Medicine, National & Kapodistrian University of Athens, Athens, GRC; 2 Department of Biomathematics, University of Thessaly School of Medicine, Larissa, GRC; 3 Neurology, Hellenic Headache Society, Athens, GRC; 4 Department of Neurology, Evaggelismos General Hospital, Athens, GRC; 5 School of Medicine, University of Thessaly, Larissa, GRC; 6 Center for Clinical Evidence Synthesis, Tufts University School of Medicine, Boston, USA; 7 Institute for Clinical Research and Health Policy Studies, Tufts Medical Center, Boston, USA; 8 Department of Nephrology, University of Thessaly School of Medicine, Larissa, GRC; 9 Neurology, General University Hospital of Larissa, Larissa, GRC

**Keywords:** anti-calcitonin gene-related peptide (cgrp) monoclonal antibodies, onabotulinumtoxina, topiramate, absolute risk difference, chronic migraine (cm), meta-analysis

## Abstract

Chronic migraine (CM) imposes significant personal, societal, and financial burdens, historically lacking specific prophylactic treatments. Monoclonal antibodies (mAbs) targeting calcitonin gene-related peptide (CGRP) represent a novel, mechanism-based, and migraine-specific prophylactic approach. Four mAbs, namely, erenumab, fremanezumab, galcanezumab, and eptinezumab, have been marketed, although head-to-head trials with standard anti-migraine treatments are absent. This study aimed to compare the efficacy and safety of anti-CGRP mAbs with standard anti-migraine treatments using a cross-trial indirect model of the absolute risk difference (ARD) of a 50% responder rate, in order to express the final results in terms of the number needed to treat (NNT) and number needed to harm (NNH). Phase 3 and 2b randomized controlled trials (RCTs) for CM prophylaxis were searched in the MEDLINE and CENTRAL databases with specific inclusion and exclusion criteria. The ARD between groups for the percentage of trial participants who reported a 50% reduction in monthly migraine days and the differences in the number of adverse events (AEs), serious adverse events (SAEs), and participants who withdrew from each RCT were calculated, and subsequently, the NNT and NNH were calculated for each one of the outcome measures. In total, eight RCTs were considered eligible. A similar efficacy and safety have been demonstrated among CGRP mAbs and all standard CM treatments. The results of the ARD for the total number of studies concerning efficacy, total adverse events, serious adverse events, and dropout from the RCTs ranged from -0.688 (95% confidence interval (CI): -0.821-(-0.513)) to -0.018 (95% CI: -0.044-(0.007)), from 0.032 (95% CI: -0.041, 0.104) to -0.380 (95% CI: -0.589, -0.126), from -0.025 (95% CI: -0.046, -0.006) to 0.014 (95% CI: -0.015, 0.42), from 0.048 (95% CI: -0.112, 0.014) to 0.232 (95% CI: -0.016, 0.458) correspondingly. All anti-CGRP mAbs showed a roughly equal statistically significant ARD and similar NNTs, ranging from 5 to 8, while the ARD of onbotulinum toxin A (oBTA) was not significant with an NNT 56. The two studies of topiramate showed contradictory results, the one significant while the other not, with NNTs 2 and 22, respectively. All four anti-CGRP mAbs showed an invariably high efficacy among their studies, in terms of the ARD and its derivative measure of NNT, in contrast to oBTA, while in topiramate, the results are contradictory between the two studies.

## Introduction and background

Migraine is a chronic, polygenetic, and disabling disease characterized by recurrent moderate to severe headaches with specific accompanying symptoms. It has a global annual prevalence of 14.7% [1]. Based on the headache frequency, migraine is classified into episodic and chronic types. Chronic migraine (CM) is defined by headaches occurring ≥15 days per month for ≥3 months, with migraine features present on at least eight days per month. By contrast, episodic migraine (EM) involves headaches occurring on <15 days per month. Globally, about 1-2% of the population suffers from CM, with a prevalence 2.5-6.5 times higher in women (1.7-4.0%) than in men (0.6-0.7%) [2]. The therapeutic approach to migraine involves both the treatment of acute phases and the prevention of migraine attacks, categorized into symptomatic and prophylactic strategies. Both pharmacological and non-pharmacological modalities (such as neurostimulation devices and nutritional supplements) are included. Symptomatic pharmacological treatments include medications like paracetamol, non-steroidal anti-inflammatory drugs (NSAIDs), triptans, antiemetics, and opioids [3]. Prophylactic treatments are crucial for both EM and CM, as they help reduce the number of migraine days and prevent the progression from EM to CM [4].

Prophylactic treatment for migraine is initiated based on specific criteria, including frequent migraine episodes (usually more than four days per month) that affect the patient’s daily activities, overuse of acute headache medications, and special conditions such as hemiplegic migraine. The decision to start prophylactic treatment is made collaboratively with the patient, considering their preferences, lifestyle, and medical history. Various drug interventions are used for CM, although not all are recommended by official guidelines. Among the older generation of prophylactic pharmacological treatments for CM, topiramate is the only one supported by relatively reliable randomized controlled trials (RCTs). In all European countries and the United States (US), topiramate is recommended as a first-line treatment for CM. Although it is not officially approved for this use, its recommendation is based on its efficacy demonstrated in two RCTs. One of these trials had a very small number of participants. Nonetheless, topiramate is favored primarily due to its low cost [5-6].

Since 2013, onbotulinum toxin A (oBTA) has been the only treatment officially indicated for CM, with significantly better quality of evidence than topiramate, supported by two double-blind RCTs [7-9]. The latest additions to the prophylactic treatment options for both EM and CM are the anti-CGRP mAbs. CGRP is a neuropeptide composed of 37 amino acids and is the most potent vasodilator in the human body. Intravenous infusion of CGRP induces migraine attacks in most sufferers [7]. CGRP receptors are located in the perivascular endings of the Aδ nerve fibers of the trigeminal nerve branches, as well as in the spinal cord, gastrointestinal tract, musculoskeletal system, heart, and blood vessels. During a migraine attack, CGRP levels significantly increase, particularly in the cerebral vasculature, as evidenced by blood samples from the jugular vein, and also in the systemic circulation [10]. Given the crucial role of CGRP in migraine pathophysiology, anti-CGRP mAbs were developed as a therapeutic target for migraine prophylaxis [11].

To date, the following four mAbs have been approved by the United States (US) and European authorities (Food and Drug Administration (FDA) and European Medicines Agency (EMA)): eptinezumab, erenumab, fremanezumab, and galcanezumab. All of these are administered subcutaneously on a monthly basis, except for eptinezumab, which is administered intravenously every three months. In addition, fremanezumab, although typically administered subcutaneously each month, can also be given every three months. RCTs have been published for each of these four mAbs, covering both EM and CM [12-16].

In many European countries and the US, anti-CGRP mAbs are recommended when traditional prophylactic treatments have proven ineffective, particularly in cases of high-frequency EM or CM, characterized by nine or more migraine days per month. These medications have demonstrated satisfactory efficacy in RCTs for both EM and CM. The convenience of self-administration at home and the generally favorable tolerability profile make them appealing options. However, their main drawback is their high cost [4].

The objective of this meta-analysis is to evaluate the ARD across four key outcome measures in RCTs comparing mAbs targeting CGRP or its receptor with established treatments for CM available prior to the introduction of anti-CGRP mAbs (e.g., oBTA, topiramate). The outcome measures under scrutiny include a) efficacy, quantified as a 50% reduction in migraine days; b) incidence of adverse events; c) occurrence of serious adverse events; and d) rate of dropouts in the RCTs. By comparing these parameters, we aim to provide a comprehensive assessment of the relative efficacy and safety profiles of anti-CGRP mAbs in comparison to conventional CM treatments.

## Review

Methods

Literature Search

For this meta-analysis, we adhered to the guidelines outlined in the Preferred Reporting Items for Systematic Reviews and Meta-Analyses (PRISMA) 2020 protocol. We conducted a thorough search of RCTs involving mAbs, such as eptinezumab, erenumab, fremanezumab, and galcanezumab, as well as oBTA and topiramate. Our search was carried out using the MEDLINE databases and the Cochrane Library’s Cochrane Central Register of Controlled Trials (CENTRAL). We employed the search term "chronic migraine" across all fields, with the following limiting filters: a) article type: "randomized controlled trial," b) language: "English," and c) species: "human." Upon retrieval of RCTs, their titles and abstracts underwent meticulous scrutiny. Selection criteria were based on predefined eligibility and exclusion criteria. In instances where initial screening did not conclusively determine the relevance to our study, full texts were obtained for further evaluation. Given that the diagnosis of CM was established in the International Classification of Headache Disorders II (ICHD-II) in 2004, our search spanned from January 1, 2004, to December 31, 2020. 

The following keywords were used: ((migraine) OR (chronic migraine)) AND ((Eptinezumab) OR (ALD403)), ((migraine) OR (chronic migraine)) AND ((Galcanezumab) OR (LY2951742)), ((migraine) OR (chronic migraine)) AND ((Fremanezumab) OR (TEV-48125)), ((migraine) OR (chronic migraine)) AND ((Erenumab) OR (AMG334)), ((migraine) OR (chronic migraine)) AND (OnabotulinumtoxinA) OR (Botox)), ((migraine) OR (chronic migraine)) AND (Topiramate).

Selection of Randomized Controlled Trials

The RCTs selected involved patients receiving at least two different interventions.

Selection Criteria

The selection of RCTs adhered to specific criteria. The included studies were phase 2b and above, encompassed patients diagnosed with CM, and those who were categorized as RCTs employing a parallel-group design, wherein the participants were randomly allocated to two or more intervention groups. Each trial featured at least one experimental intervention group receiving the active medication and one group receiving a placebo.

Exclusion Criteria

RCT studies were excluded if they involved other types of headaches, such as trials investigating mAbs for cluster headaches. In addition, studies published in languages other than English were excluded. Furthermore, study protocols, post-hoc analyses, subgroup analyses of RCTs, small pilot RCT studies, and all non-RCT study designs were also excluded from the analysis.

Outcome Measures

The ARD in the efficacy of pharmacological interventions, defined as a 50% reduction in migraine days compared to baseline, was selected as the primary outcome measure. In addition, the ARD in adverse effects and serious adverse events of the drug interventions, along with the ARD in clinical trial participants who dropped out, were included in the study. Key statistical terminologies are summarized in Table 1 for reference.

**Table 1:** Statistical terminologies

Statistical terms	Definitions
Relative risk	It is the probability that an individual will develop a disease while exposed to an agent divided by the probability that an individual will develop the disease while not exposed to the agent. Relative risk expresses the probability of a disease occurring in one group of the population relative to another group of the population.
Absolute risk	It expresses the probability of the disease occurring in a specific group of the population.
Risk difference	Risk difference or absolute risk reduction is the difference between the risk of developing a disease in those who are exposed to the agent minus the risk of developing the disease in those who are not exposed to the agent. It expresses the additional risk that a person who has been exposed to the agent will develop the disease, compared to a person who has not been exposed to the agent. "ARD _efficacy_= AR_ efficacy placebo _– AR_ efficacy drug_ " "ARD _adverse event_= AR_ adverse event placebo _– AR_ adverse event drug_" "ARD _serious adverse event_ = AR_ serious adverse event_ – AR_ serious adverse event drug _" "ARD _drop out_= AR_ drop out placebo _– AR_ drop out drug_"
Number needed to treat	It is the number of people who need to be treated in order to prevent an additional event. It is a derivative size of the absolute risk difference and in particular its inverse size (NNT= 1/RD). In recent years, in the field of migraine, in the effort to integrate the patient into the therapeutic treatment decision, the use of this specific quantity is increasingly observed, as it is fairly understandable and does not require specialized knowledge of statistics. In fact, in 2020 and 2021, for the newest anti-CGRP mAbs, systematic analyses and meta-analyses have been published, using NNT and RD, in order to highlight their effectiveness and side effects.

Extraction of Results and Statistical Analysis

After the final selection of studies, data were extracted and recorded using Excel® version 16.56 (Microsoft Corporation, Redmond, Washington, USA). Meta-analysis was performed with StatsDirect® version 3.2.10 (StatsDirect Ltd., Wirral, UK) software.

For each study, the following information was recorded: title, authors, date of publication, study population, effectiveness of pharmacological interventions (expressed as the percentage of patients who experienced at least a 50% reduction in migraine days per month, known as the 50% response rate), number of adverse events, number of serious adverse events, and number of withdrawals from the RCTs.

The following statistical measures were calculated and used to create forest plots: a) absolute risk difference of effectiveness, based on the 50% response rate (percentage of patients experiencing at least a 50% reduction in migraine days), b) absolute risk difference in the total number of adverse events, c) absolute risk difference in the number of serious adverse events, and d) absolute risk difference in the number of dropouts from the RCTs.

The I² statistic was used to assess heterogeneity between studies. The risk of bias in the RCTs was evaluated using the ROB 2 tool, as recommended by the Cochrane Handbook for Systematic Reviews [17].

The decision to study the ARD and its derivative measures, NNT and NNH, aims to assess the efficacy and side effects of both new and existing CM pharmacological treatments. This approach aligns with recent literature on the NNT measure [18,19]. These metrics are easy to understand and do not require specialized statistical knowledge, allowing the general public to comprehend them and engage in treatment decisions with their physicians. In addition, these measures are valuable for the pharmaceutical industry, regulatory authorities (Food and Drug Administration (FDA) and European Medicines Agency (EMA)), and insurance funds in determining the approval and reimbursement of new pharmacological interventions.

Results

Selection of Meta-Analysis Literature

**Figure 1:**
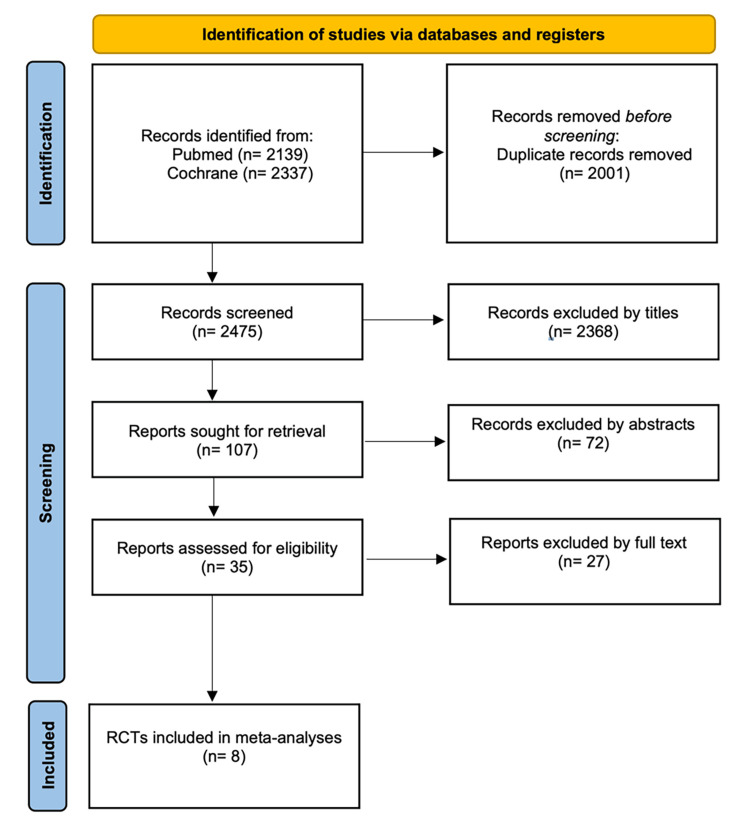
Flow chart of the literature searched.

Literature selection was performed in three steps, as presented in the PRISMA 2020 flow chart (Figure 1). The initial search in Pubmed and the Cochrane Library identified 4.476 publications, which were screened by title and abstract. A total of 2,001 publications were excluded because they were duplicates in the two databases. Then, based on the reading of the titles, 2,440 publications were excluded from the abstracts because a) they were of a different topic, b) they did not concern intervention, c) they were secondary analyses, d) they were subanalyses, e) they were case reports, and f) they were RCT protocols. The remaining 27 were assessed based on full publication. The final number of studies included in the meta-analysis was eight, of which five studies were related to mAbs, one study related to oBTA, and two studies related to topiramate.

Characteristics of Meta-Analysis Studies

Table 2 presents the baseline characteristics of the studies included in the meta-analysis. The total number of participants across the studies was 5,924, with the mean age of participants ranging from 37.8 to 47.8 years old. Gender distribution was predominantly female across all subpopulations.

**Table 2:** Characteristics of the studies CM: chronic migraine, HD: headache days, MD: migraine days, MHD: monthly headache days, MMD: monthly migraine days, RCT: randomized controlled trial, SD: standard deviation

Author, year	Study design	Duration	Inclusion criteria	Sample size	Drug (mg)	Sex (male/female)	Mean age (SD)
Silberstein et al., 2020 [12]	RCT phase 3	24 weeks	CM >12 months, ≥15 to ≥​​26 HD, ≥8 MD	1072	Eptinezumab (100)	49/307	41 (11.7)
Silberstein et al., 2020 [12]	RCT phase 3	24 weeks	CM >12 months, ≥15 to ≥26 HD, ≥8 MD	1072	Eptinezumab (300)	16/314	41 (10.4)
Silberstein et al., 2020 [12]	RCT phase 3	24 weeks	CM >12 months, ≥​​​​​​​15 to ≥​​​​​​​26 HD, ≥​​​​​​​8 MD	1072	Placebo	41/325	40.5 (11.2)
Tepper et al., 2017 [13]	RCT phase 2	12 weeks	≥​​​​​​​15 MMD	667	Erenumab (70)	25/166	41.4 (11.3)
Tepper et al., 2017 [13]	RCT phase 2	12 weeks	≥​​​​​​​15 MMD	667	Erenumab (140)	30/160	42.9 (11.1)
Tepper et al., 2017 [13]	RCT phase 2	12 weeks	≥​​​​​​​15 MMD	667	Placebo	60/226	42.1 (11.3)
Detke et al., 2018 [14]	RCT phase 3	12 weeks	≥​​​​​​​15 MMD ≥8 MMD	1113	Galcanezumab (120)	51/226	41.1 (12.4)
Detke et al., 2018 [14]	RCT phase 3	12 weeks	≥​​​​​​​15 MMD ≥8 MMD	1113	Galcanezumab (240)	73/483	41.6 (12.1)
Detke et al., 2018 [14]	RCT phase 3	12 weeks	≥​​​​​​​15 MMD ≥8 MMD	1113	Placebo	73/483	41.6 (12.1)
Mulleners et al., 2020 [15]	RCT phase 3	12 weeks	≥​​​​​​​4 MMD, 2-4 failure migraine preventive medication	193	Galcanezumab (120)	12/83	45.8 (11.6)
Mulleners et al., 2020 [15]	RCT phase 3	12 weeks	≥​​​​​​​4 MMD, 2-4 failure migraine preventive medication	193	Placebo	13/85	44.8 (13.1)
Silberstein et al., 2017 [16]	RCT phase 3	12 weeks	≥​​​​​​​15 MHD	1130	Fremanezumab (675)	45/331	42 (12.4)
Silberstein et al., 2017 [16]	RCT phase 3	12 weeks	≥​​​​​​​15 MHD	1130	Fremanezumab (225)	49/330	40.6 (12)
Silberstein et al., 2017 [16]	RCT phase 3	12 weeks	≥​​​​​​​15 MHD	1130	Placebo	45/330	41.4 (12)
Dodick et al., 2010 [9]	RCT phase 3	32 weeks	≥​​​​​​​15 MHD ≥50% migraines	1384	OnabotulinumtoxinA	85/603	41.1 (11.8)
Dodick et al., 2010 [9]	RCT phase 3	32 weeks	≥​​​​​​​15 MHD ≥50% migraines	1384	Placebo	103/593	41.5 (9.5)
Diener et al., 2007 [5]	RCT phase2	20 weeks	≥​​​​​​​15 MHD	59	Topiramate (100)	8/24	47.8 (9.4)
Diener et al., 2007 [5]	RCT phase2	20 weeks	≥​​​​​​​15 MHD	59	Placebo	7/20	44.4 (9.6)
Silberstein et al., 2007 [6]	RCT phase 2	16 weeks	≥​​​​​​​15 MHD	306	Topiramate (100)	25/128	37.8 (12.38)
Silberstein et al., 2007 [6]	RCT phase 2	16 weeks	≥​​​​​​​15 MHD	306	Placebo	20/133	38.6 (11.80)

Efficacy of mAbs

**Figure 2:**
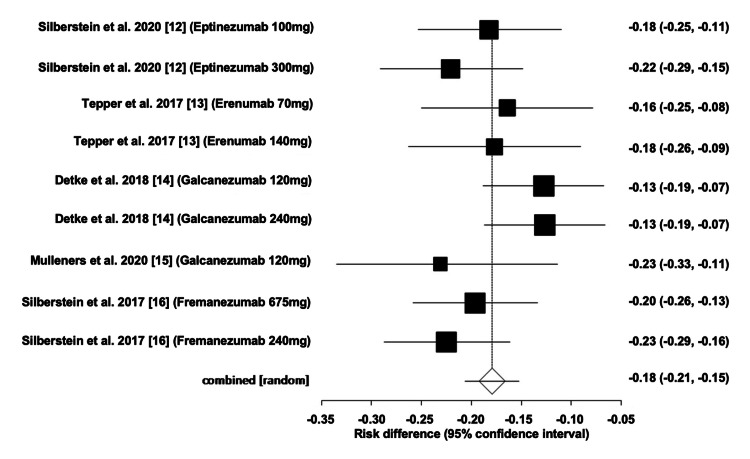
Forest plot of absolute risk difference of migraine monoclonal antibody efficacy.

**Figure 3:**
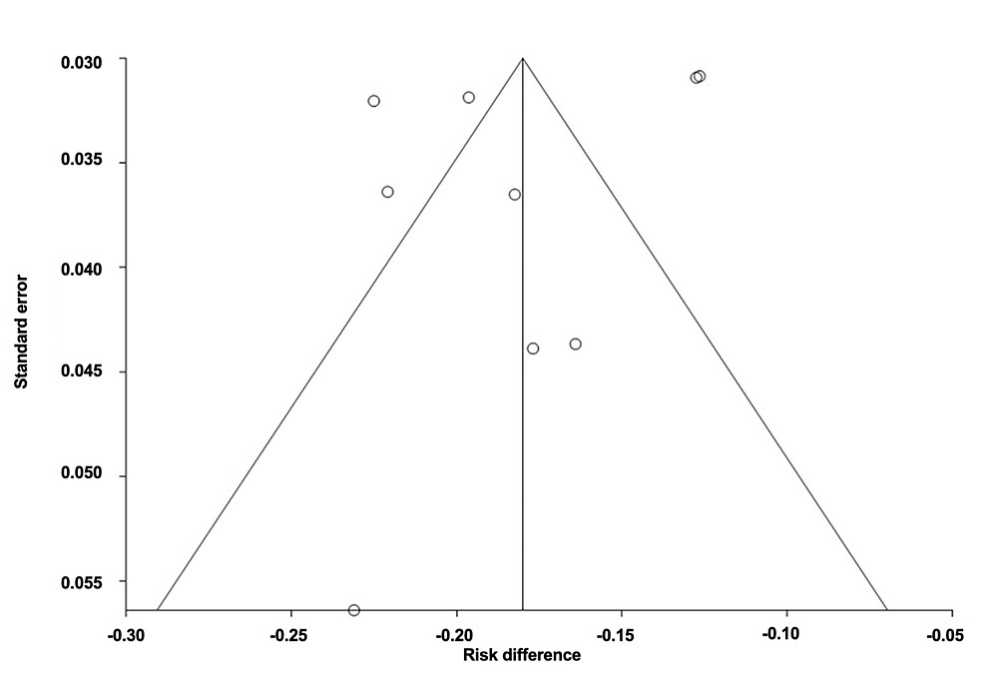
Funnel plot of absolute risk difference of migraine monoclonal antibody efficacy.

The overall ARD for the efficacy of mAbs was estimated to be -0.18 (95% CI: -0.210, -0.15), corresponding to a number needed to treat (NNT) of 6. Heterogeneity, as indicated by I^2^, was found to be 22.70%, with PQ = 0.24 and PE = 0.36. Figures 2-3 illustrate the forest and funnel plots, respectively.

Adverse Events of mAbs

**Figure 4:**
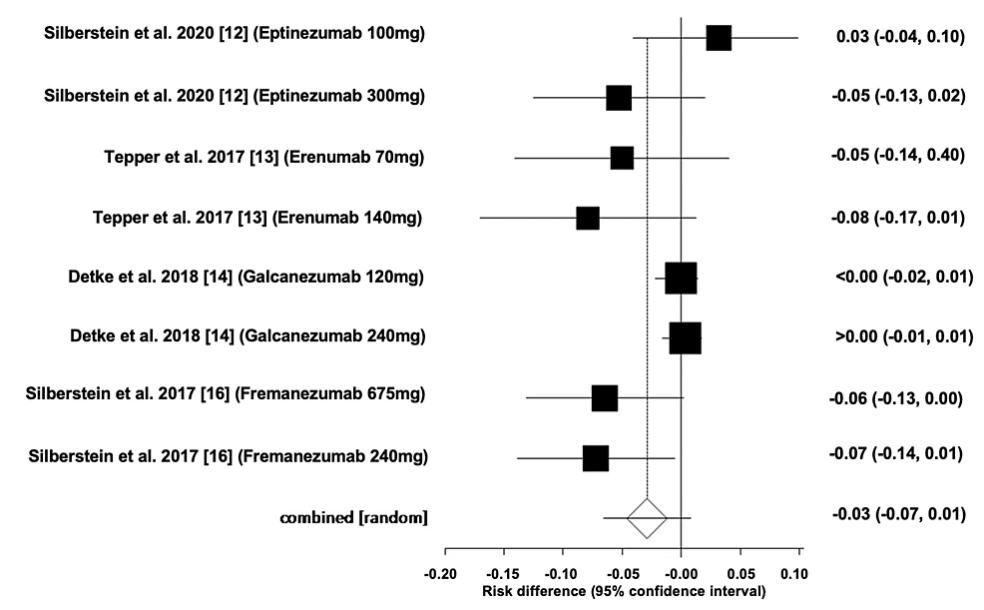
Forest plot of the absolute risk difference of migraine mAbs.

**Figure 5:**
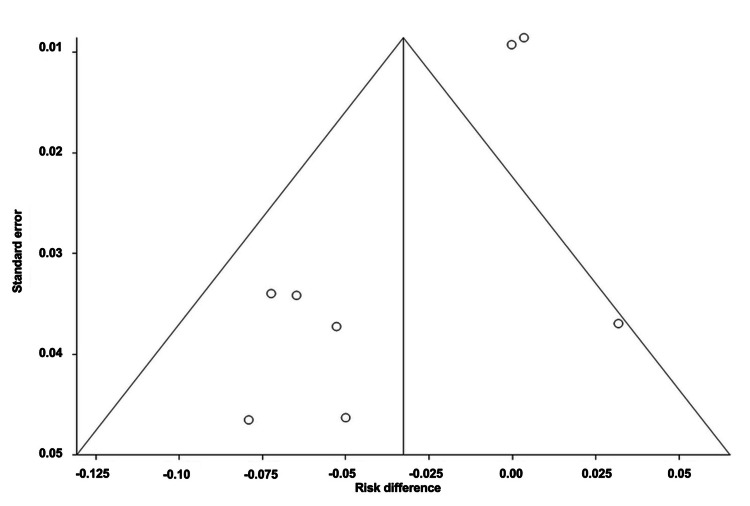
Funnel plot of the absolute risk difference of migraine mAbs.

The pooled ARD for the total adverse events associated with mAbs was estimated to be -0.03 (95% CI -0.07, 0.01). Heterogeneity, denoted by I^2^, was found to be 86.90%, with PQ < 0.0001 and PE = 0.03. Figures 4-5 illustrate the forest and funnel plots, respectively.

Serious Adverse Events of mAbs

**Figure 6:**
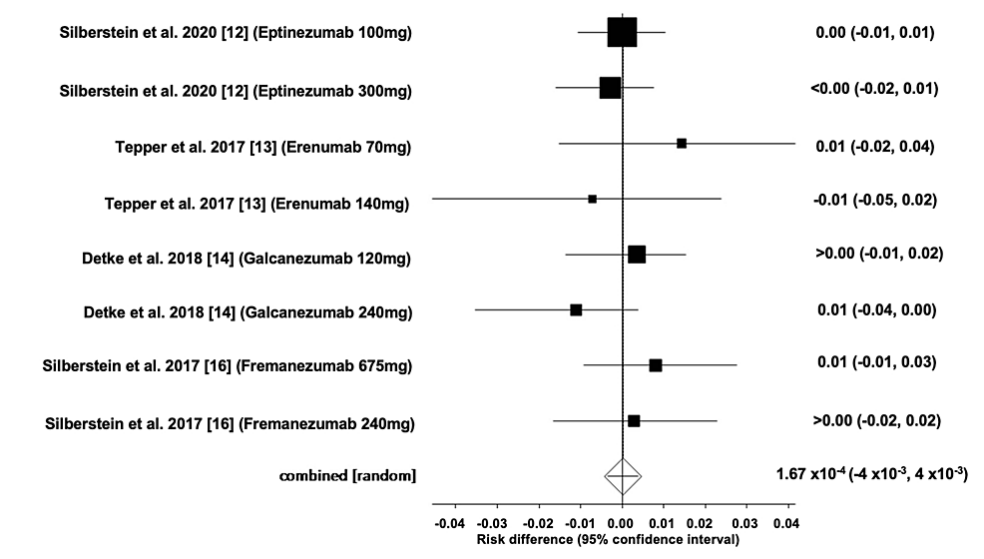
Forest plot of the absolute risk difference of serious adverse events of migraine mAbs.

**Figure 7:**
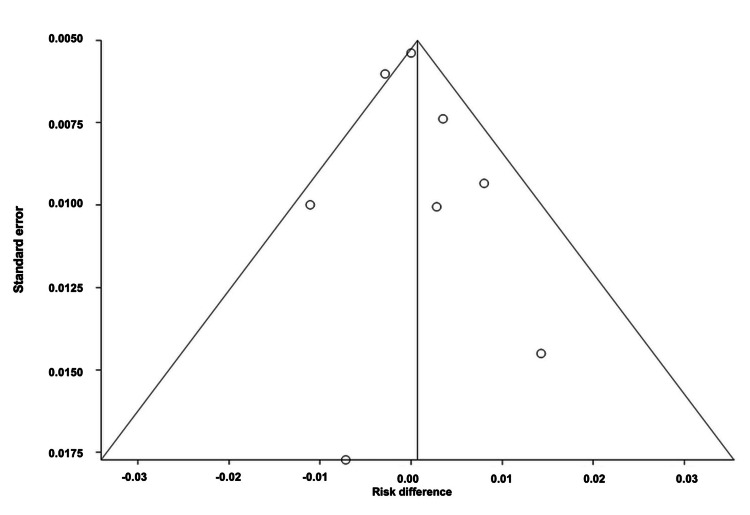
Funnel plot of the absolute risk difference of serious adverse events of migraine mAbs.

The pooled ARD for serious adverse events (AEs) associated with mAbs was estimated to be 0.0001 (95% CI: -0.004, 0.004). Heterogeneity, represented by I^2^, was found to be 0.00%, with PQ = 0.60 and PE = 0.69. Figures 6-7 illustrate the forest and funnel plots, respectively.

Number of Participants Who Dropped Out of RCTs of mAbs Due to Adverse Events

**Figure 8:**
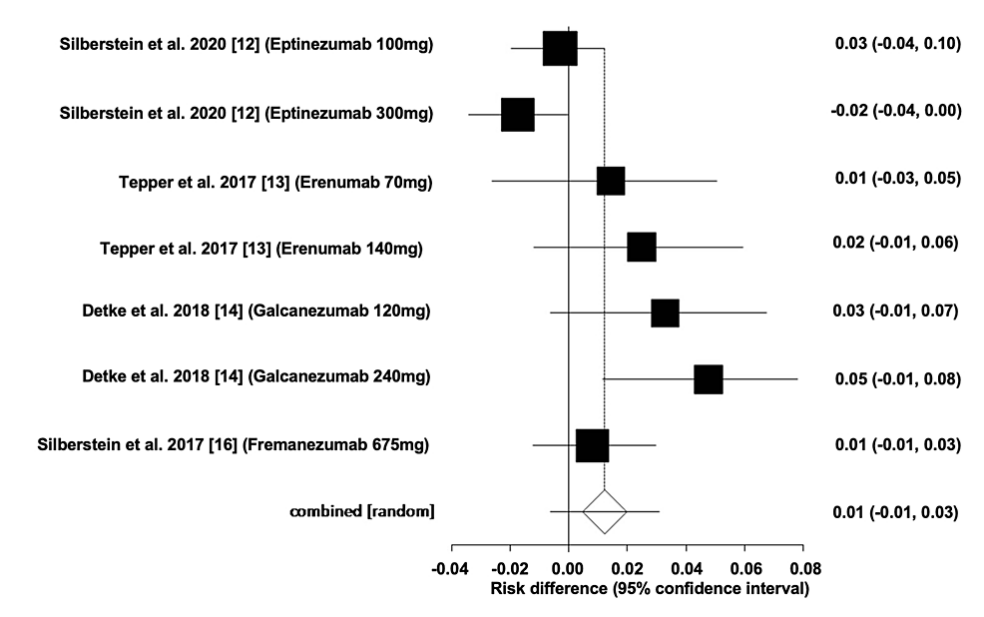
Forest plot of the absolute risk difference of the number of participants who dropped out of the RCTs of migraine mAbs.

**Figure 9:**
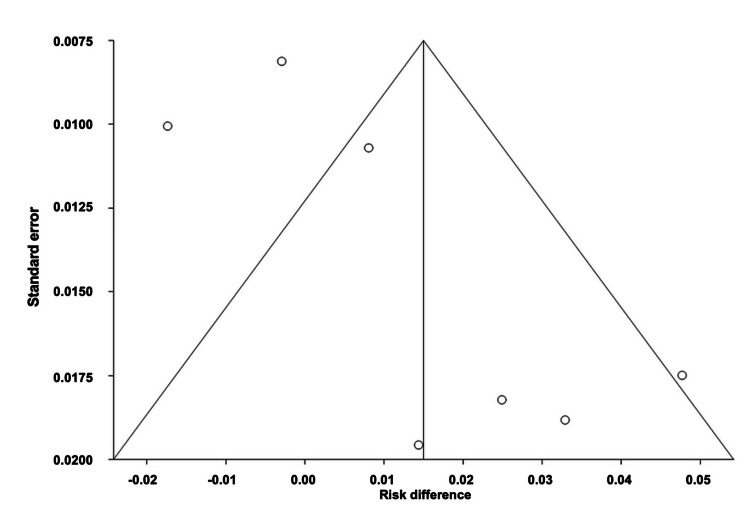
Funnel plot of the absolute risk difference of the number of participants who dropped out of the RCTs of migraine mAbs.

The overall ARD for the number of participants who dropped out of the RCTs of mAbs was estimated to be 0.01 (95% CI: -0.01, 0.03). Heterogeneity, indicated by I^2^, was found to be 78.0%, with PQ = 0.0001 and PE = 0.03. Figures 8-9 illustrate the forest and funnel plots, respectively.

Comparison of the Effectiveness of All Interventions

**Figure 10:**
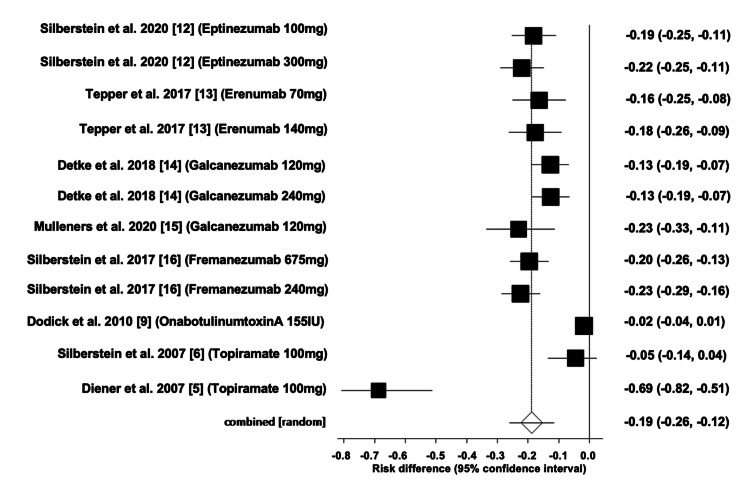
Forest plot of the absolute risk difference of the effectiveness of all interventions.

**Figure 11:**
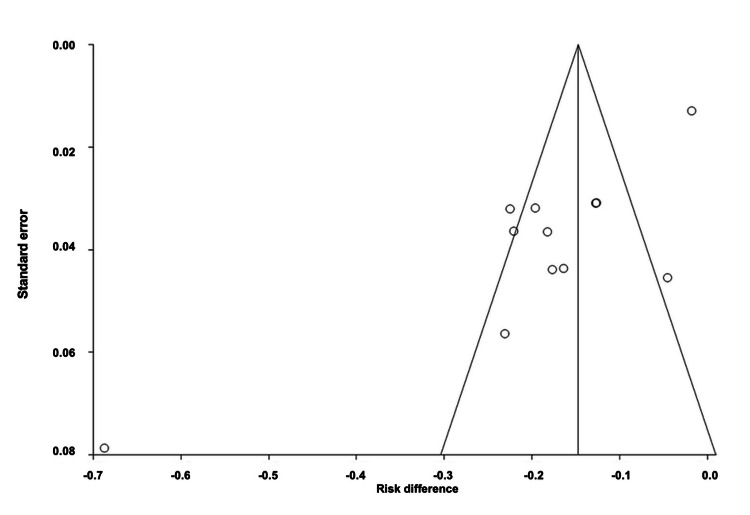
Funnel plot of the absolute risk difference of the effectiveness of all interventions.

The pooled ARD for the effectiveness of all interventions was estimated to be 0.188, with a 95% CI of (-0.26, -0.11). Heterogeneity, as indicated by I^2^, was found to be 93.4%, with PQ < 0.0001 and PE = 0.0005. Figures 10-11 illustrate the forest and funnel plots, respectively.

Comparison of Adverse Events of All Interventions

**Figure 12:**
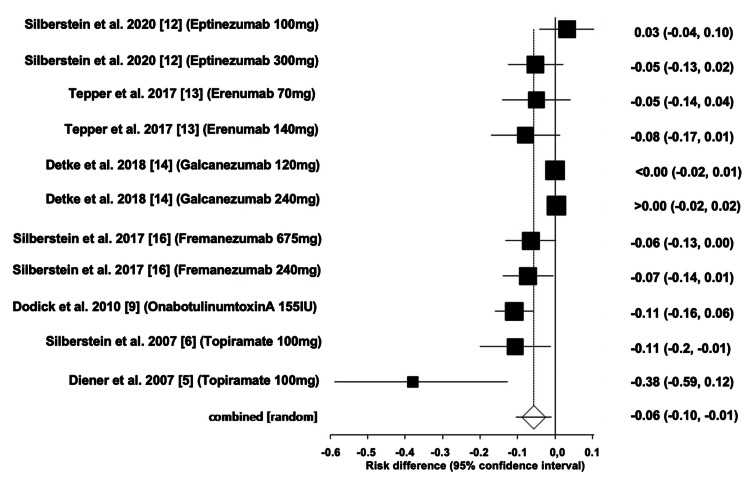
Forest plot of the absolute risk difference of adverse events of all interventions.

**Figure 13:**
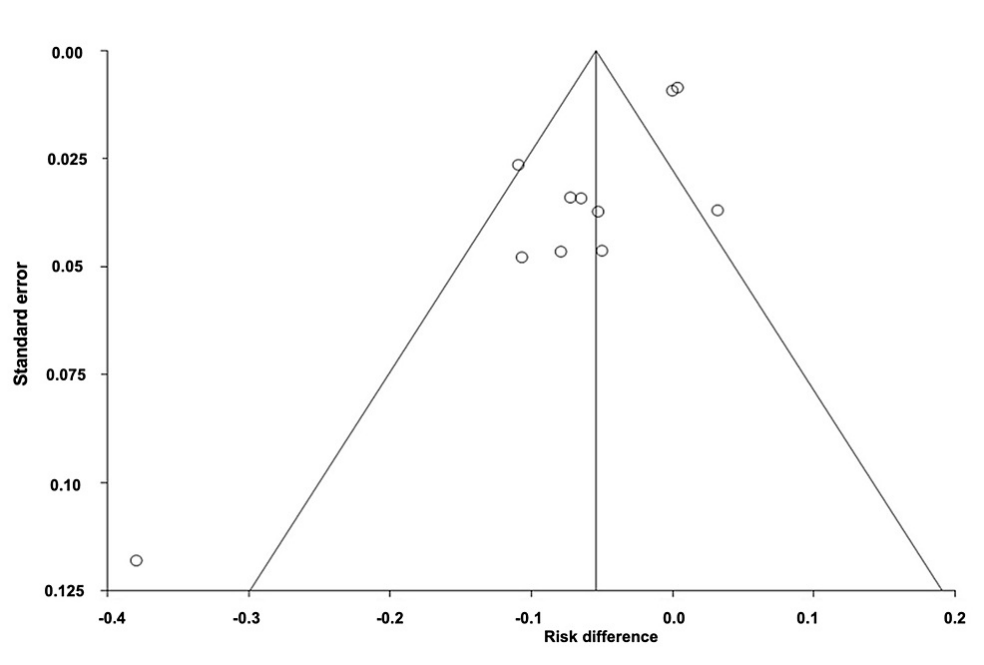
Funnel plot of the absolute risk difference of adverse events of all interventions.

The pooled ARD for total AEs of all drug interventions was estimated to be -0.06 (95% CI: -0.10, -0.01). Heterogeneity, as measured by I^2^, was found to be 92.90%, with PQ < 0.0001 and PE = 0.0035. Figures 12-13 illustrate the forest and funnel plots, respectively.

Comparison of Serious Adverse Events of All Drug Interventions

**Figure 14:**
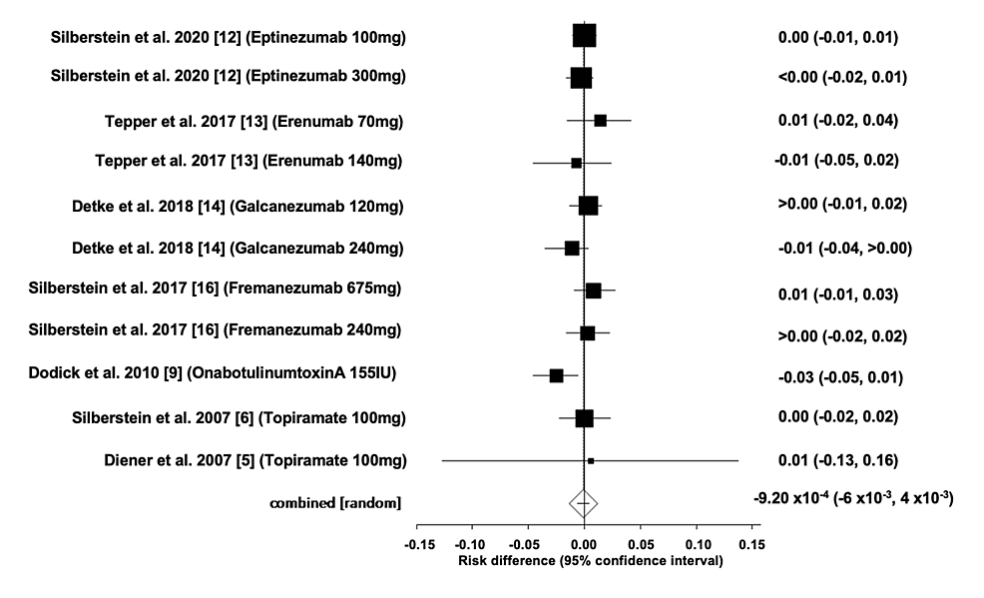
Forest plot of the absolute risk difference of serious adverse events of all drug interventions.

**Figure 15:**
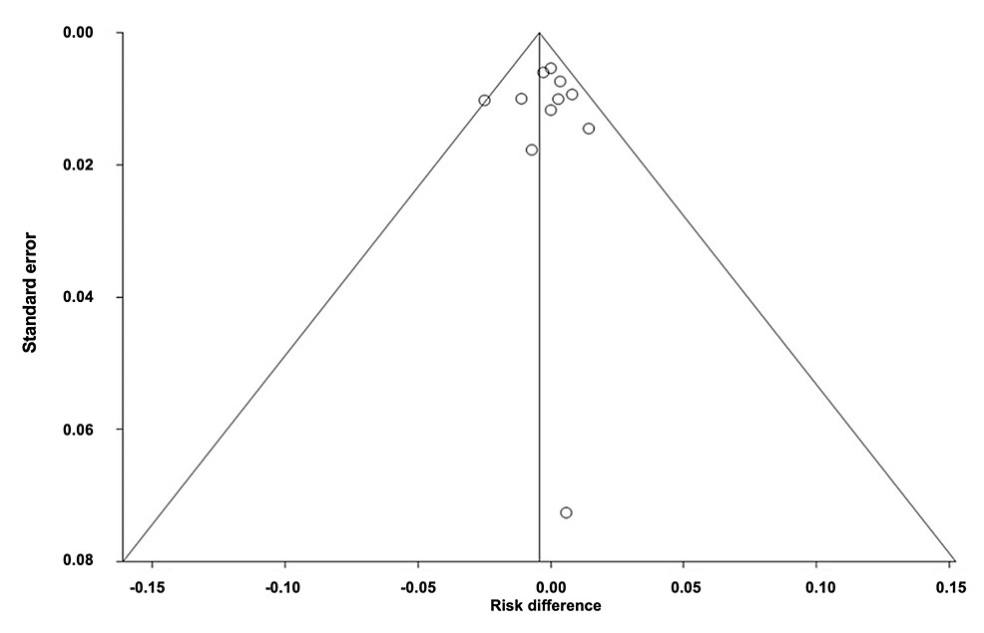
Funnel plot of the absolute risk difference of serious adverse events of all drug interventions.

The overall ARD for serious AEs of all drug interventions was estimated to be -0.0009 (95% CI: -0.006, 0.004). Heterogeneity, as measured by I^2^, was found to be 37.10%, with PQ = 0.10 and PE = 0.92. Figures 14-15 depict the forest and funnel plots, respectively.

Comparison of the Number of Participants Who Dropped Out From the RCTs of All Interventions

**Figure 16:**
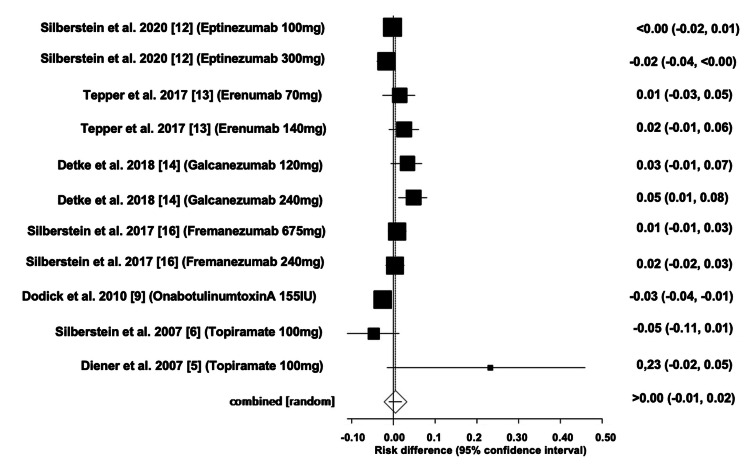
Forest plot of the absolute risk difference of the number of participants who dropped out of the RCTs of all interventions.

**Figure 17:**
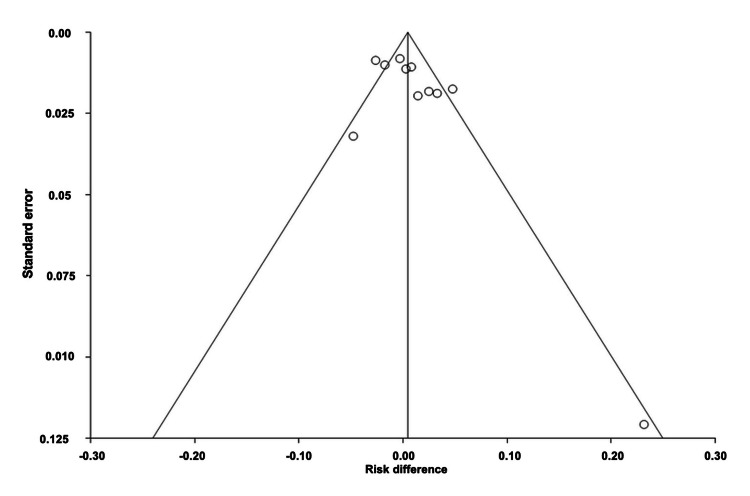
Funnel plot of the absolute risk difference of the number of participants who dropped out of the RCTs of all interventions.

The overall difference in the number of participants who dropped out of the RCTs of all interventions was estimated to be 0.004 (95% CI: -0.01, 0.019). Heterogeneity, as measured by I^2^, was found to be 74.10%, with PQ < 0.0001 and PE = 0.075. Figures 16-17 depict the forest and funnel plots, respectively.

Comparison of the NNT of All Interventions

In Table 3 and Table 4, the NNT and NNH of all interventions are presented and calculated using the risk difference (RD) formula (NNT = 1/RD). Since a negative NNT or NNH value is not meaningful, the absolute value of RD is used in the formula 1/RD for the calculation of NNH.

**Table 3:** NNT of all interventions calculated by the risk difference of efficacy. NNT: number needed to treat

Trial ID	Treatment	NNT	95% confidence interval
Silberstein et al., 2020 [12]	Eptinezumab 100 mg	6	(4,10)
Silberstein et al., 2020 [12]	Eptinezumab 300 mg	5	(4,7)
Tepper et al., 2017 [13]	Erenumab 70 mg	7	(4,13)
Tepper et al., 2017 [13]	Eremanezumab 140 mg	6	(4,12)
Detke et al., 2018 [14]	Galcanezumab 120 mg	8	(1,15)
Detke et al., 2018 [14]	Galcanezumab 240 mg	8	(1,15)
Silberstein et al., 2017 [16]	Fremanezumab 675 mg	6	(4,7)
Silberstein et al., 2017 [16]	Fremanezumab 240 mg	5	(4,7)
Dodick et al., 2010 [9]	OnabotulinumtoxinA 155 IU	56	(-∞,23) (142,+∞)
Diener et al., 2007 [5]	Topiramate 100 mg	2	(2,2)
Silberstein et al., 2007 [6]	Topiramate 100 mg	22	(-∞,8) (23,+∞)

**Table 4:** NNH of all interventions calculated by the risk difference of adverse events. NA: non-applicable, NNH: number needed to harm, AE: adverse event, SAE: serious adverse event

Trial ID	Treatment	NNH _AE_	95% Confidence Interval	NNH _SAE_	95% Confidence Interval	NNH _dropouts_	95% Confidence Interval
Silberstein et al., 2020 [12]	Eptinezumab 100 mg	32	(-∞,-25)(10, +∞)	NA	NA	334	(-∞,-52)(82 +∞)
Silberstein et al., 2020 [12]	Eptinezumab 300 mg	19	(-∞,-8)(48, +∞)	334	(-∞,-63)(125, +∞)	58	(26,5000)
Tepper et al., 2017 [13]	Erenumab 70 mg	20	(-∞,-8)(25,+∞)	72	(-∞,-67)(24,+∞)	70	(-∞,-39)(20,+∞)
Tepper et al., 2017 [13]	Eremanezumab 140 mg	13	(-∞,-6)(82,+∞)	143	(-∞,-7)(42,+∞)	41	(-∞,-85)(17,+∞)
Detke et al., 2018 [14]	Galcanezumab 120 mg	5000	(-∞,-46)(70,+∞)	250	(-∞,-72)(67,+∞)	31	(-∞,-157)(15,+∞)
Detke et al., 2018 [14]	Galcanezumab 240 mg	286	(-∞,-62)(58,+∞)	91	(-∞,-29)(250,+∞)	21	(13,87)
Silberstein et al., 2017 [16]	Fremanezumab 675 mg	16	(-∞,-8)(417,+∞)	125	(-∞,-112)(38,+∞)	125	(-∞,-83)(34,+∞)
Silberstein et al., 2017 [16]	Fremanezumab 240 mg	14	(8,179)	334	(-∞,-59)(44,+∞)	345	(-∞,-53)(40,+∞)
Dodick et al., 2010 [9]	OnabotulinumtoxinA 155 IU	10	(7,18)	40	(22,167)	39	(23,95)
Diener et al., 2007 [5]	Topiramate 100 mg	3	(8,17)	167	(-∞,-8)(6,37,+∞)	5	(-∞,-64)(3,+∞)
Silberstein et al., 2007 [6]	Topiramate 100 mg	10	(5,80)	NA	NA	21	(-∞,-9)(75,+∞)

Risk of Bias of Meta-Analysis Studies

**Figure 18:**
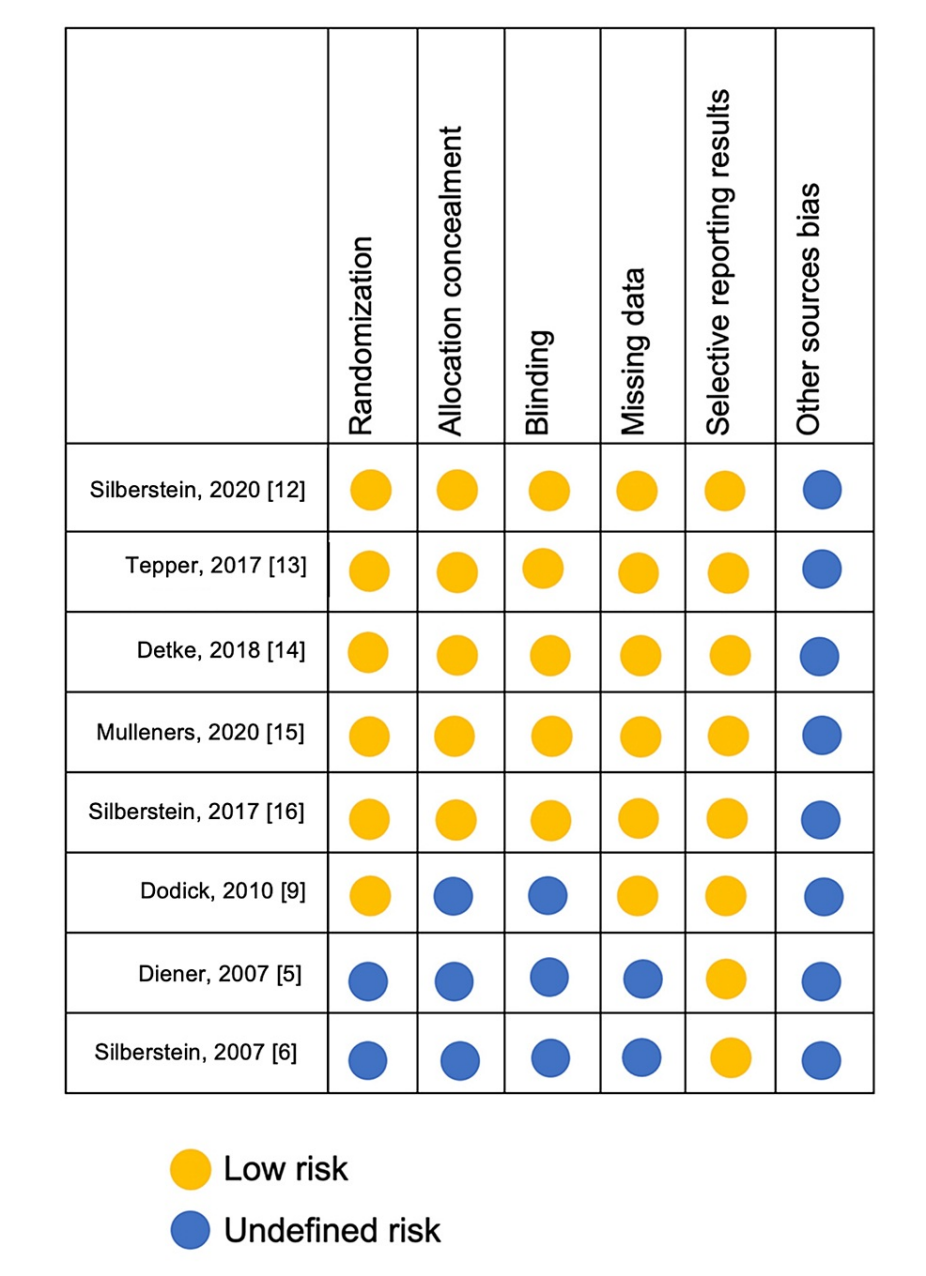
Risk of bias of meta-analysis studies.

According to the Cochrane Handbook of Systematic Reviews, the risk of bias of the studies was assessed and is presented in Figure 18.

Discussion

In this study, we conducted a meta-analysis focusing on newer migraine medications, specifically anti-CGRP mAbs. These medications represent a novel therapeutic approach and have undergone extensive testing in RCTs, targeting both EM and CM. The findings from these trials have demonstrated promising efficacy and excellent safety profiles [12-16]. Migraine ranks as the second leading cause of disability worldwide, according to the Global Burden of Disease [1]. Given the significant burden of this condition, the development of new treatments is of paramount importance and holds promise for improving patient outcomes. However, it is essential to acknowledge the high cost associated with these specific interventions. Therefore, continuous re-evaluations of efficacy and side effects are imperative to optimize decision-making regarding their administration.

Similar Efficacy and Adverse Events Among CGRP mAbs

It appears that across the four measures, namely, effectiveness, AEs, SAEs, and dropouts-anti-CGRP mAbs, did not exhibit significant differences. This conclusion is drawn from the observation that the confidence intervals for these measures overlapped and intersected the ensemble line. This suggests that there were no substantial variations in these outcomes among the different anti-CGRP mAbs studied.

Similar Effectiveness and Adverse Events Comparing CGRP mAbs and Older Drug Interventions

The efficacy comparison between anti-CGRP mAbs and the currently utilized interventions in CM, namely, oBTA and topiramate, revealed interesting findings. Notably, in the efficacy comparison, there was an overlap of the confidence intervals among anti-CGRP mAbs, oBTA, and topiramate [6]. However, a difference was observed in the topiramate study [5], which may be attributed to the very small sample size (N = 59). Despite this, comparisons of adverse events, serious adverse events, and the number of participants who withdrew from the RCTs showed no significant differences among the interventions.

The discrepancy observed in the NNT values for oBTA between the pooled results of the two PREEMPT studies and the ARD meta-analysis underscores the importance of employing methodologically robust meta-analytic techniques for indirect comparisons among RCTs. This emphasizes the need to avoid numerical comparisons among independent studies without a systematic approach.

It is indeed worth highlighting that while the NNT analysis conducted through ARD meta-analysis focuses solely on the binary variable of the 50% responder rate, the continuous variable of mean monthly migraine days may provide additional insights. In the case of oBTA, the mean monthly migraine days are numerically very similar compared to anti-CGRP mAbs, contrasting with the ARD of the 50% responder rate and the resulting NNT. Therefore, it is imperative to adopt a comprehensive critical perspective, considering all major efficacy outcome measures, especially in cases where there are discrepancies. This approach helps prevent potentially misleading conclusions regarding the efficacy of the drug under study and ensures a more thorough understanding of its clinical impact.

It is noteworthy that eptinezumab and fremanezumab exhibited the lowest NNT values in their respective studies. However, it is essential to recognize that while there may be numeric differences in the NNT values among different interventions, these differences may not be substantial, especially if the confidence intervals overlap. The overlapping confidence intervals suggest that the observed differences in NNT values are not statistically significant, indicating that there is no clear distinction in efficacy among eptinezumab, fremanezumab, and other anti-CGRP mAbs. Therefore, despite the numeric variation in NNT values, the overall efficacy of these medications appears to be comparable.

The significant difference in NNT values observed between the two studies of topiramate, namely, 2 and 22, is indeed striking. This difference is further emphasized by the vastly different sample sizes, which are 59 and 306 participants, respectively. It is important to note that a pooled analysis of the raw data from these studies is not available, in contrast to the case of oBTA. The smaller study with a sample size of 59 participants and an NNT of 2 is clearly underpowered, potentially leading to unreliable or exaggerated estimates of treatment effects. Conversely, the larger study with a sample size of 306 participants is likely to provide more robust and valid results. Given the discrepancies in NNT values and sample sizes between the two topiramate studies, caution is warranted when interpreting their findings. The larger study is likely to be more reliable, but further research and replication of results are necessary to confirm the true efficacy of topiramate in the treatment of migraine.

Regarding the NNH for all AEs, all four mAbs exhibited AEs comparable to placebo, with NNH values ranging from 13 to 5,000. The vast majority of these AEs were local reactions at the injection sites. By contrast, the NNH values for oBTA and topiramate were ≤10, indicating a significant statistical difference compared to placebo. However, it is important to note that all these adverse events were reversible and temporary. These findings suggest that while mAbs have a generally favorable safety profile with AEs comparable to placebo, oBTA and topiramate may have a higher incidence of AEs. Nonetheless, the reversible and temporary nature of these AEs should be considered when evaluating the overall safety and tolerability of these treatments.

It is notable that the rarest serious adverse events occurred with eptinezumab and monthly fremanezumab, with a calculated NNH of 334 for both drugs. Galcanezumab followed closely with an NNH of 250, while erenumab and topiramate presented the lowest NNH values. However, it is important to recognize that while there may be numeric differences in the NNH values among different drugs, these differences may not be statistically significant or clinically meaningful. In other words, the observed variations in NNH values do not indicate a significant difference in the risk of serious adverse events between these medications. Therefore, despite the differences in NNH values, it is essential to interpret these findings cautiously and consider them in the context of the overall safety profile of each medication. Further research and larger studies may be needed to confirm these findings and assess the clinical significance of any observed differences.

The results regarding the NNH for dropouts were very similar across the different medications. Although there were numeric differences in NNH values, these differences did not reach statistical significance, with the exception of a marginal significance for 240 mg galcanezumab (indicating fewer adverse events than placebo) and onabotulinumtoxinA (oBTA, indicating more adverse events than placebo). However, these differences may not be clinically meaningful. It is essential to interpret these findings cautiously and within the broader context of the study. Numeric differences in NNH values without statistical significance suggest that the risk of dropouts associated with these medications is generally comparable. Therefore, further research is necessary, alongside careful consideration of other factors, such as efficacy, tolerability, and patient preferences, to make well-informed decisions about the use of these medications in clinical practice. This comprehensive approach will ensure that treatment decisions are optimized for the best patient outcomes.

Limitations

Important limitations have emerged in this study. The use of ARD and NNT for comparisons between RCTs assumes homogeneity regarding the design of the studies and the characteristics of the participants. While the RCTs included in the meta-analysis were generally similar in terms of design, demographics, and clinical characteristics of participants, there may still be sources of bias due to inherent heterogeneity. In addition, the populations included in RCTs may differ from those encountered in daily clinical practice, potentially limiting the generalizability of the findings.

Furthermore, the majority of RCTs had relatively short durations, typically three to six months, which may not fully capture the longer-term effectiveness of the interventions. It is worth noting that adverse events, including serious adverse events, of a drug can manifest after it has been placed on the market, and the incidence and severity of adverse events may be more accurately assessed in post-marketing surveillance.

In addition, the RCT conducted by Mulleners et al. (2020) was included only in the meta-analysis of efficacy and not in the analysis of other outcome measures, as the published results concerning the excluded measures had pooled data from both episodic migraine and CM patients.

Finally, an important limitation concerns the high rates of heterogeneity observed in some measures in the meta-analysis, particularly in adverse events and the number of participants who dropped out of RCTs of mAbs. This heterogeneity makes it challenging to draw definitive conclusions. Specifically, a large degree of heterogeneity was observed in adverse events and dropout rates of RCTs involving mAbs compared with effectiveness, adverse events, and dropouts from RCTs of all drug interventions.

Future Directions

Indeed, it is imperative to conduct "head-to-head" RCTs that directly compare the therapeutic benefits of different interventions in terms of clinically important endpoints. In addition, cost-effectiveness studies for these interventions are essential. However, conducting such studies poses challenges due to their high cost and the large number of patients required to recognize the equivalence, inferiority, or superiority of one treatment option over another.

By conducting head-to-head RCTs, the neurology community can make more informed decisions regarding the appropriate administration of specific drugs. Furthermore, the results of these studies can inform healthcare policymakers and insurance providers, enabling them to make decisions about covering these drugs through social security funds or other reimbursement mechanisms. Ultimately, such comprehensive research efforts are necessary to optimize patient care and resource allocation in the management of migraine and other neurological conditions.

## Conclusions

The NNT is indeed a valuable outcome measure for effectively communicating results to patients and insurance agencies. It provides a clear understanding of the treatment benefit by indicating the number of patients needed to be treated for one additional patient to experience the desired outcome, such as a 50% responder rate. Performing a risk difference meta-analysis to calculate NNTs based on the binary outcome of a 50% responder rate allows for direct comparison among interventions. In the case of anti-CGRP mAbs, they demonstrated very similar and favorable NNT values. However, there was a contrast observed with oBTA, suggesting differences in treatment effectiveness. In addition, the results of topiramate were contradictory between the two studies, indicating a need for further investigation and consideration when making treatment decisions.
